# *Listeria monocytogenes* Transmission from Donated Blood to Platelet Transfusion Recipient, Italy

**DOI:** 10.3201/eid2910.230746

**Published:** 2023-10

**Authors:** Maria Gori, Luca Bolzoni, Erika Scaltriti, Lilia Andriani, Vito Marano, Francesca Morabito, Clara Fappani, Danilo Cereda, Enza Giompapa, Rosa Chianese, Paola Lanzini, Livia Antonietta Martinelli, Silvia Bianchi, Antonella Amendola, Stefano Pongolini, Elisabetta Tanzi

**Affiliations:** Università degli Studi di Milano, Milan, Italy (M. Gori, C. Fappani, S. Bianchi, A. Amendola, E. Tanzi);; Istituto Zooprofilattico Sperimentale della Lombardia e dell'Emilia-Romagna (IZSLER), Parma, Italy (L. Bolzoni, E. Scaltriti, S. Pongolini);; Azienda Socio Sanitaria Territoriale della Valtellina e dell’Alto Lario, Sondrio, Italy (L. Adriani, V. Marano, F. Morabito, P. Lanzini);; Directorate General for Health, Lombardy Region, Milan (D. Cereda);; Agenzia di Tutela della Salute della Montagna, Sondrio (E. Giompapa, L.A. Martinelli);; Agenzia Regionale Emergenza Urgenza, Milan (R. Chianese);; Azienda Socio Sanitaria Territoriale dei Sette Laghi, Varese, Italy (R. Chianese)

**Keywords:** Listeria monocytogenes, bacteria, listeriosis, whole-genome sequencing, bloodborne transmission, surveillance, Italy

## Abstract

We report *Listeria monocytogenes* infection in a patient in Italy who was transfused with pooled platelet concentrate. Genomic analysis revealed that *L. monocytogenes* isolates from the donor blood unit, the transfused platelets, and the patient’s blood culture were genetically closely related, confirming transfusion transmission. Additional surveillance and secondary bacterial screening could improve transfusion safety.

*Listeria monocytogenes* is a gram-positive, primarily foodborne pathogen responsible for severe invasive infections (*1*), especially in immunocompromised patients. The treatment for some immunodeficiency conditions can require the administration of blood products, which pose additional risks for patients’ health, although not generally connected with *L. monocytogenes* bacteremia. One case of transfusion-transmitted *L. monocytogenes* infection has been reported in the literature (*2*). Conversely, 2 case reports describe platelet products contaminated by *L. monocytogenes*, but the contamination was intercepted before transfusion (*3*,*4*). We describe a case of transfusion-related *L. monocytogenes* infection in a patient who received a pooled-platelet concentrate.

## The Study

On June 13, 2022, a 78-year-old woman was admitted to an emergency department in the Lombardy Region of northern Italy, reporting persistent fever, nausea, and vomiting. According to her medical history, she had gastric adenocarcinoma and had undergone total gastrectomy with splenectomy on February 15, 2021, and postoperative anemia was subsequently observed. In September 2021, she experienced cancer progression in the liver and, beginning in March 2022, she underwent chemotherapy with trifluridine/tipiracil.

At the time of her emergency admission, hematological tests revealed a severe pancytopenia, likely due to chemotherapy. Her hemoglobin level was 8 g/dL (reference range 12–16 g/dL), platelet count was 27,000/µL (reference range 150,000–450,000/µL), procalcitonin was 1.51 ng/mL (reference range 0.00–0.50 ng/mL), prothrombin time was 1.33 (reference range 0.80–1.20), activated partial thromboplastin time was 0.66 (reference range 0.80–1.20), and C-reactive protein was 318.7 mg/L (reference range 0.0–5.0 mg/L). Clinicians administered granulocyte growth factor therapy, transfusion treatment with concentrated red blood cells, and empirical antibiotic therapy with piperacillin-tazobactam. Blood cultures incubated in the BD BACTEC FX system (Becton Dickinson, https://www.bd.com) showed no growth.

On June 14, 2022, the patient underwent a transfusion of buffy coat–pooled platelet derived from 5 different donors. After transfusion of ≈150 mL of platelet products, the woman experienced chills, nausea, and fever of 37.8°C. Treatment was stopped and hydrocortisone was administered, based on the diagnosis of transfusion reaction. The adverse reaction was reported to the Italian National Blood Centre on June 20, 2022. Antibiotic therapy was boosted with meropenem. A second blood culture yielded positive results 25 hours after collection, and *L. monocytogenes* was identified by matrix-assisted laser desorption/ionization time-of-flight mass spectrometry (Bruker Daltonics, https://www.bruker.com). 

The isolate was susceptible to ampicillin, erythromycin, meropenem, penicillin, and trimethoprim/sulfamethoxazole. In response to this finding, on June 15, 2022, antibiotic therapy was adjusted to ampicillin/sulbactam and gentamicin and was continued for 21 days. Within 24 hours of adjusting antimicrobial drug therapy, the patient was afebrile. Because no additional complications were reported during hospitalization, the patient was discharged on July 5, 2022, hemodynamically stable. The woman died of progressive cancer on October 22, 2022.

We tested each of the 5 donated platelet units for bacterial contamination by using the BacT/ALERT 3D system (bioMérieux, https://www.biomerieux.com). We detected contamination in a single-donor unit, which we subsequently cultured and found positive for *L. monocytogenes*. The other blood component units obtained from the same unit (donated on June 9, 2022) were destroyed, and no other patients were transfused from them. The donor was recalled on June 16, 2022, but, because of quarantine for COVID-19, did not arrive at the transfusion center until June 28, 2022. The donor was investigated for possible risk factors related to the bacterial infection, such as consumption of contaminated food (none were identified) and confirmed the absence of gastrointestinal or febrile symptoms at the time of donation. Despite consideration of the time elapsed since donation, blood was drawn for cultures; results were negative.

The *L. monocytogenes* isolates recovered from the buffy coat platelet concentrate, the transfused patient’s blood cultures, and the single-donor unit were sent to the regional reference laboratory (RRL) of Lombardy Region, Italy. On the basis of Ministry of Health provisions, RRLs in Italy perform whole-genome sequencing (WGS) to characterize *L. monocytogenes* isolates (*5*). We extracted genomic DNA by using the Maxwell HT 96 gDNA Blood Isolation System (Promega, http://www.promega.com) and conducted WGS on the Nextseq 550 system (Illumina, http://www.illumina.com). We performed in silico sequence type (ST) and PCR serogroup detection by using Institut Pasteur’s BIGSdb-*Lm* (*6*), which revealed that the 3 isolates belonged to ST1 and PCR serogroup 4b. We performed comparative genomics to analyze allele distances in core-genome multilocus sequence typing (cgMLST) by using Pasteur’s cgMLST allelic scheme (*6*) and analyzed SNPs by using the CFSAN SNP Pipeline version 2.1.1 (*7*). We observed neither allelic nor SNP differences. Four other ST1 clinical strains isolated in Lombardy Region in 2022 showed distances of 0–1 allele in cgMLST and 0–2 SNPs differences to the isolates involved in the transfusion-associated case. We used the strain originated from the donated blood unit (BioSample no. ERS15898914) as a reference for SNP analysis. The size of the core alignment between all 7 genomes was 2,930,365 bp, 98.7% of the reference strain’s length. Trace-back investigation revealed that no other patients from the 2022 Lombardy outbreak had a history of blood transfusion. Moreover, epidemiologic investigation forms of the infected patients did not identify any food products as a common source of infection.

## Conclusions

Current guidelines in transfusion medicine in Italy recommend testing for hepatitis B and C viruses, HIV, and serologic markers of *Treponema pallidum*. Mandatory tests do not consider bacterial contamination (*8*). Although both gram-positive and gram-negative organisms have reportedly caused transfusion-associated sepsis (*9*), the use of pathogen-reduction technology is not mandatory and was not applied on the platelets transfused to our patient.

Contamination of blood products commonly occurs because of introduction of skin microbiota at the phlebotomy site or donations by asymptomatic donors with transient bacteremia (*9*,*10*). The environment also can be a source of contamination, particularly when bag containers are breached during transfusion product processing, transport, or storage (*11*). Bacterial contamination is a higher risk in platelet products because platelets must be stored at room temperature to maintain viability and function, which could facilitate the growth of a wide spectrum of bacteria. Approximately 1 of every 1,000–3,000 platelet units are estimated be contaminated with bacteria (*12*–*14*).

In the case we described, bloodborne transmission was strongly supported by WGS-based typing, which confirmed that the *L. monocytogenes* isolates recovered from the transfused patient’s blood cultures, the platelet concentrate, and the blood donor’s platelet unit were genetically closely related. Although the source of the contamination was unclear, one explanation could be environmental contamination of the donated blood unit. Several cases of contamination of transfusion products by environmental bacteria have been described (*12*). *L. monocytogenes* is widespread in the environment and could have entered the blood unit via undetected defects or damages in the storage container. Another explanation could be a transient bacteremia experienced by the donor who was asymptomatic at the time of donation, such as in a previously described case (*2*). Questionnaires administered to assess blood donors’ health and medical history are not useful to identify *L. monocytogenes* infection because healthy persons will generally be asymptomatic. Indeed, persons with transient asymptomatic bacteremia, or who are in the recovery phase of an infection, still can qualify as blood donors.

In conclusion, bacterial contamination of donated blood and blood components still represents a major public health issue globally. This case highlights the need to improve safety in transfusion medicine by implementing surveillance activities and additional measures, such as secondary testing and pathogen-reduction technology.
